# Cryptic signals: substomatal architecture influences stomatal responses to red light and CO_2_

**DOI:** 10.1111/nph.70667

**Published:** 2025-10-21

**Authors:** Muhammad Haroon, Caroline Ivsic, Frances C. Sussmilch, Anju Manandhar, Scott A. M. McAdam

**Affiliations:** ^1^ Department of Botany and Plant Pathology Purdue University West Lafayette IN 47907 USA; ^2^ School of Biological Sciences University of Western Australia Crawley WA 6009 Australia; ^3^ School of Natural Sciences University of Tasmania Sandy Bay TAS 7005 Australia

**Keywords:** blue light, guard cells, mesophyll, protective cells, Restionaceae

## Abstract

- The mesophyll provides a critical signal for stomatal responses to red light (RL) and CO_2_ in angiosperms. By contrast, the stomatal response to blue light (BL) is largely guard cell‐specific. It is not known whether substomatal or mesophyll anatomy influences the effectiveness of the mesophyll signal driving stomatal responses to RL and CO_2_.
- Here we utilize the diverse substomatal anatomy in Restionaceae to investigate whether mesophyll anatomy has an influence on stomatal responses to light and CO_2_. Restionaceae from the subfamily Restionoideae have distinctive nonphotosynthetic, cuticle‐covered protective cells that line a large substomatal cavity, while most species from the subfamily Leptocarpoideae have a small substomatal cavity surrounded by mesophyll cells.
- We found that representative Restionoideae species do not have a stomatal response to RL or CO_2_, with only BL driving stomatal responses to light. By contrast, representative Leptocarpoideae species have a stomatal response to RL, BL and CO_2_.
- The absence of stomatal responses to RL and CO_2_ in species with protective cells lining the substomatal cavity demonstrates the importance of mesophyll anatomy in stomatal control and suggests that the mesophyll signal that drives stomatal responses to RL and CO_2_ requires close proximity of photosynthetic cells to the guard cells.

## सारांश

- मेसोफिलले एंजियोस्पर्महरूमा रातो प्रकाश (RL) र CO_2_ मा स्टोमेटल प्रतिक्रियाहरूको लागि एक महत्वपूर्ण संकेत प्रदान गर्दछ। यसको विपरित, नीलो प्रकाश (BL) मा स्टोमेटल प्रतिक्रिया धेरै हदसम्म गार्ड सेल‐विशिष्ट हुन्छ। यो थाहा छैन कि सबस्टोमेटल वा मेसोफिल एनाटॉमीले RL र CO_2_ मा स्टोमेटल प्रतिक्रियाहरू चलाउने मेसोफिल सिग्नलको प्रभावकारितालाई प्रभाव पार्छ।
- यहाँ हामी प्रकाश र CO_2_ मा स्टोमेटल प्रतिक्रियाहरूमा मेसोफिल एनाटॉमीको प्रभावकारिता छ कि छैन भनेर अनुसन्धान गर्न रेस्टोनेसीमा विविध सबस्टोमेटल एनाटॉमी प्रयोग गर्छौं। उपपरिवार रेस्टोनेसीबाट रेस्टोनेसीमा विशिष्ट गैर‐फोटोसिंथेटिक, क्यूटिकल‐ढाकिएको सुरक्षात्मक कोशिकाहरू हुन्छन् जुन ठूलो सबस्टोमेटल गुहालाई रेखांकित गर्छन्, जबकि उपपरिवार लेप्टोकार्पोइडीका धेरैजसो प्रजातिहरूमा मेसोफिल कोशिकाहरूले घेरिएको सानो सबस्टोमेटल गुहा हुन्छ।
- हामीले पत्ता लगायौं कि प्रतिनिधि रेस्टोनेसी प्रजातिहरूमा RL वा CO_2_ मा स्टोमेटल प्रतिक्रिया हुँदैन, केवल BL ले प्रकाशमा स्टोमेटल प्रतिक्रियाहरू चलाउँछ। यसको विपरित, प्रतिनिधि लेप्टोकार्पोइडी प्रजातिहरूमा RL, BL र CO_2_ प्रति स्टोमेटल प्रतिक्रिया हुन्छ।
- सबस्टोमेटल गुहामा रहेका सुरक्षात्मक कोषहरू भएका प्रजातिहरूमा RL र CO_2_ प्रति स्टोमेटल प्रतिक्रियाहरूको अनुपस्थितिले स्टोमेटल नियन्त्रणमा मेसोफिल शरीर रचनाको महत्त्वलाई प्रदर्शन गर्दछ र सुझाव दिन्छ कि RL र CO_2_ प्रति स्टोमेटल प्रतिक्रियाहरू चलाउने मेसोफिल संकेतलाई गार्ड कोषहरूसँग प्रकाशसंश्लेषक कोषहरूको नजिकको आवश्यकता पर्दछ।

- मेसोफिलं एन्जियोस्पर्मय् रेड लाइट (RL) व CO_2_या स्तोमेटल प्रतिक्रियाया निंतिं छगू महत्वपूर्ण संकेत बी। थ्व स्वया, ब्लु लाइट (बिएल)या स्टोमेटल प्रतिक्रिया यक्व ल्याखय् गार्ड सेल‐विशिष्ट जुइ। सबस्टोमेटल वा मेसोफिल एनातोमीं आरएल व सीओ२या स्टोमेटल प्रतिक्रियायात न्ह्याकीगु मेसोफिल सिग्नलया प्रभावकारितायात प्रभावित याइ कि मयाइ धका सीके मफुनि।
- थन जिमिसं रेस्टियोनेसीइ विविध सबस्टोमेटल एनातोमीया छ्येला मेसोफिल एनातोमीया प्रकाश व सीओ २ या स्टोमेटल प्रतिक्रियाय् प्रभाव दु कि मदु धकाः अनुसन्धान याना। रेस्टियोनोइदेया उपपरिवारया रेस्टियोनेसीइ विशिष्ट गैर‐प्रकाशसिन्थेटिक, क्यूटिकलं त्वपुयातःगु सुरक्षात्मक कोष दइ गुकिलिं तःधंगु सबस्तोमेटल क्याभिटी दइ धाःसा उपपरिवार लेप्टोकार्पोइदेया अप्व प्रजातिइ मेसोफिल कोषं घेरेयानातःगु चिधंगु सबस्टोमेटल क्याभिटी दइ।
- जिमिसं प्रतिनिधि रेस्टियोइडे प्रजातिइ आरएल वा सीओ २ या स्तोमेटल प्रतिक्रिया मदुगु खने दु, गुकिलि बीएल जक्क प्रकाशया स्तोमेटल प्रतिक्रिया चलेयाइ। थ्व स्वया प्रतिनिधि लेप्टोकार्पोइडिया प्रजातिइ आरएल, बीएल व सीओ२या निंतिं स्टोमेटल प्रतिक्रिया दइ।
- सबस्टोमेटल क्याभिटीइ सुरक्षात्मक कोष दूगु प्रजातिइ आरएल व सीओ२या स्तोमेटल प्रतिक्रिया मदुगुलिं स्तोमेटल नियन्त्रणय् मेसोफिल एनातोमीया महत्त्व क्यनि व आरएल व सीओ२या स्तोमेटल प्रतिक्रियायात न्ह्याकीगु मेसोफिल संकेतयात फोटोसिन्थेटिक कोषया निकटता माःगु सुझाव बी।

## Résumé

- Le mésophylle fournit un signal déterminant pour les réponses stomatiques à la lumière rouge (LR) et au CO_2_ chez les angiospermes. À l’inverse, la réponse stomatique à la lumière bleue (LB) est en grande partie spécifique des cellules de garde. On ignore si l’anatomie de la chambre sous‐stomatique ou celle du mésophylle influence l’efficacité du signal mésophyllien qui pilote les réponses stomatiques à la LR et au CO_2_.
- Ici, nous exploitons la diversité de l’anatomie de la chambre sous‐stomatique chez les Restionacées afin d’examiner si l’anatomie du mésophylle influe sur les réponses stomatiques à la lumière et au CO_2_. Les Restionacées de la sous‐famille des Restionoïdées possèdent des cellules protectrices distinctives, non photosynthétiques et recouvertes de cuticule, qui bordent une large chambre sous‐stomatique, tandis que la plupart des espèces de la sous‐famille des Leptocarpoïdées présentent une petite chambre sous‐stomatique entourée de cellules mésophyllienne.
- Nous avons constaté que des espèces représentatives des Restionoïdées n’exhibent pas de réponse stomatique à la LR ni au CO_2_, la LB étant la seule à déclencher des réponses stomatiques à la lumière. À l’inverse, des espèces représentatives des Leptocarpoïdées présentent une réponse stomatique à la LR, à la LB et au CO_2_.
- L’absence de réponses stomatiques à la LR et au CO_2_ chez les espèces dont la chambre sous‐stomatique est bordée de cellules protectrices démontre l’importance de l’anatomie du mésophylle dans le contrôle stomatique et suggère que le signal mésophyllien pilotant les réponses stomatiques à la LR et au CO_2_ requiert une proximité étroite entre les cellules photosynthétiques et les cellules de garde.

## خلاصہ

- میزوفل خلیے انجیو اسپرمز پودوں میں سرخ روشنی اور کاربن ڈائ آکسائیڈ کے زیر اثر سٹومیٹل ردعمل یا کاموں کے لئے ایک اہم اشارہ بھیجتے ہیں۔ اس کے برعکس ، نیلی روشنی کا اسٹومیٹل ردعمل بڑی حد تک سٹومیٹا کے اندر گارڈز سیل یا حفاظتی خلیے سے منسلک ہے جسکا میزوفل یا اس سے نکلنے والےاشارے سے کوئ تعلق نہیں ہے۔ 
- لیکن یہ معلوم نہیں ہے مختلف طرح کی سٹوماٹا یا میزوفل کی بناوٹیں اگر سرخ روشنی اور کاربن ڈائ آکسائیڈ کی موجودگی میں اس میزوفل سے نکلنے والےاشارے پر منحصر سٹومیٹا کے ردعمل یا کام کی تاثیر کو متاثر کرتیں ہیں یا نہیں۔ یہاں ہم اس بات کی تفتیش کے لئیے ریسٹیونیسی انواع میں موجود مختلف قسم کی سٹومیٹا اور میزوفل بناوٹوں کا استعمال کرتے ہیں جو پہلے سے ان کے اندر ہیں کہ آیا یہ روشنی اور کاربن ڈائ آکسائیڈپر منحصرسٹومیٹل ردعمل پر اثر و رسوخ رکھتا ہے۔
- ریسٹیوناسی فیملی میں موجود ذیلی فیملی ریسٹیونائیڈی کی انواع میں مخصوص غیر فوٹوسینتھیٹک ، پوست یا کیوٹیکل سے ڈھکے ہوئے محافظ خلئیے یا پروٹیکٹو خلئیے ہوتے ہیں جو سٹومیٹا کی نچلی سطح سے لیکر میزوفل تک پھیلے ہوتے ہیں۔ اسی ریسٹیوناسی فیملی میں موجود ایک اور ذیلی فیملی لیپٹوکارپائڈائ کی انواع میں سٹومیٹا اور میزوفل کے درمیان حفاظتی خلیے تو موجود نہیں ہوتے لیکن تھوڑی خلا ضرور موجود ہوتی ہے۔ ہم نے یہ دریافت کیا ہے نمائندہ ریسٹیونائڈی انواع میں سرخ روشنی اور کاربن ڈائئ اکسیڈ نے کوئ سٹوماٹا کا ردعمل نہیں دکھایا بلکہ صرف نیلی روشنی کی موجودگی میں دیکھا گیا۔اس کے برعکس ، نمائندہ لیپٹوکارپائڈائی انواع میں اسٹومیٹل ردعمل کو نہ صرف سرخ روشنی بلکہ نیلی روشنی اور کاربن ڈائ آکسائیڈ کی موجودگی میں بھی دیکھا گیاہے۔
- ریسٹیونائیڈی انواع میں سٹومیٹا اور میزوفل کے درمیان حفاظتی خلیوں کی موجودگی اور سرخ روشنی اور کاربن ڈائ آکسائیڈ کے ذیر اثر سٹومیٹا کے کام کی عدم موجودگی اس بات کو ظاہر کرتی ہے کہ سرخ روشنی میں ہونے والے سٹومیٹا کے کاموں میں میزوفل کی ساخت اہمیت رکھتی ہے۔اسکے ساتھ یہ بھی تجویز کیا جاتا ہے کہ سرخ روشنی اور کاربن ڈائ آکسائیڈ کی موجودگی میں ہونے والے سٹومیٹا کے کام یا ردعمل میں میزوفل سے نکلنے والےاشارے کوموثر بنانے کے کیلئیے، سٹومیٹا اور میزوفل کا ایک دوسرے کی قربت میں ہونا ضروری ہے۔

## Introduction

Stomata are microscopic pores on the epidermis that consist of a pair of guard cells that optimize the balance between CO_2_ uptake for photosynthesis and water loss (Raschke, 1975; Lawson & Matthews, 2020). Stomata respond dynamically to a suite of environmental factors, such as light, vapor pressure deficit (VPD), CO_2_, as well as internal factors including hormones and circadian rhythms. Of these signals, stomatal responses to light are one of the most important for regulating daily photosynthesis and water use efficiency, with stomata having highly dynamic responses to diurnal light cycles, the spectral distribution of light through the day, passing clouds, sunflecks, and differential canopy cover (McCree, 1971, 1972; Grantz & Zeiger, 1986; Shimazaki *et al*., 2007; McAusland *et al*., 2016; Inoue & Kinoshita, 2017; Matthews *et al*., 2017, 2018, 2019; Lawson & Vialet‐Chabrand, 2019; Yang *et al*., 2020; Liu & van Iersel, 2021; Santos *et al*., 2021; Long *et al*., 2022; Lawson & Leakey, 2024).

In most vascular land plants, stomata open in response to increasing light intensity and close when light intensity declines (Raschke, 1975; Doi *et al*., 2015; Ivsic *et al*., 2025). Stomatal opening in the light ensures mesophyll access to atmospheric CO_2_ for photosynthesis, while closure when light intensity declines optimizes water use for carbon gain (Wong *et al*., 1979; Talbott *et al*., 2003). Blue wavelengths of light provide one of the critical light signals for stomatal responses (Shimazaki *et al*., 2007; Matthews *et al*., 2019; Vialet‐Chabrand *et al*., 2021). The blue light (BL) stomatal response in angiosperms appears to be confined to the guard cells as increased BL can directly hyperpolarize the guard cell plasma membrane, triggering stomatal opening in isolated epidermis (Zeiger & Zhu, 1998; Tominaga *et al*., 2001; Roelfsema *et al*., 2002; Inoue *et al*., 2008; Hiyama *et al*., 2017). Red wavelengths of light provide the other main light signal for stomatal responses (Roelfsema *et al*., 2002). RL‐driven stomatal opening responses can be both guard cell specific (Schwartz & Zeiger, 1984; Zhu *et al*., 2020) or require photosynthesis in the mesophyll, with an increasing fluence of RL hyperpolarizing the guard cell plasma membrane only in the presence of surrounding mesophyll cells (Roelfsema *et al*., 2002; Ando & Kinoshita, 2018). Stomatal responses to decreasing RL are only observed in intact leaves, not epidermal peels (Lee & Bowling, 1992; Lee & Bowling, 1995; Mott *et al*., 2008; McAdam & Brodribb, 2012; Fujita *et al*., 2013).

There is strong evidence that photosynthesis regulates stomatal responses (Wong *et al*., 1979) and that both photosynthesis in the guard cells and in the mesophyll is key to regulating stomatal responses to changes in RL. The mechanism linking stomatal regulation by RL via photosynthesis remains poorly understood. It has been hypothesized that the RL stomatal response is linked to both a stomatal response to internal CO_2_ (C_i_) and a C_i_‐independent response pathway, which could be linked to the redox state of plastoquinone (Taylor *et al*., 2024). Stomata respond to changes in atmospheric CO_2_ (C_a_) via C_i_, with stomata opening when C_i_ declines and closing when C_i_ increases. The stomatal response to C_a_ has been found to require intact mesophyll (Mott, 2009), although some studies show weak stomatal responses to changes in C_a_ in isolated epidermis (Lee *et al*., 2008). Recent work suggests that apoplastic sugar provides the key signal for stomatal regulation by the mesophyll (Zait *et al*., 2025). Given the apoplastic nature of the mesophyll signal for stomata, we do not yet know whether the highly diverse cellular architecture of the mesophyll (Borsuk *et al*., 2022) plays a role in the responsiveness of stomata to RL and C_a_.

To investigate whether substomatal anatomy influences stomatal responsiveness to RL and C_a_, we conducted gas exchange experiments using varying fluence rates of RL and BL, and under different C_a_. These experiments were performed on species from two subfamilies of Restionaceae that exhibit markedly divergent substomatal anatomies (Linder, 2000; Briggs & Linder, 2009; Briggs *et al*., 2014). Subfamily Restionoideae species have protective cells (Schutzzellen in the original German literature) which line a large substomatal chamber (Pfitzer, 1870; Gilg, 1891; Cutler, 1969). These cells are derived from mesophyll chlorenchyma cells but have no chloroplasts and are covered in cuticle (Pfitzer, 1870; Gilg, 1891; Linder, 2000). Being cuticle‐covered, protective cells have long been speculated to play a role in enhancing drought resistance or protecting the mesophyll from the atmosphere (Gilg, 1891; Briggs *et al*., 2014) by providing a barrier between the photosynthetic mesophyll and the guard cells. Most species from the Leptocarpoideae subfamily, with a center of diversity in Australia, lack protective cells and have common, small substomatal chambers surrounded by photosynthetic mesophyll (Briggs & Linder, 2009). This contrasting anatomy, where nonphotosynthetic and cuticle‐covered protective cells separate the mesophyll from the substomatal chamber in Restionoideae but not in most Leptocarpoideae (Briggs & Linder, 2009), provides an excellent model system for exploring how substomatal anatomy influences mesophyll‐derived stomatal signals.

We selected three representative species from Restionoideae and Leptocarpoideae, with or without protective cells lining the substomatal cavity. In these species, we measured stomatal responses to both RL and BL, separately and in combination; we also examined stomatal responses to C_a_ in a species from each subfamily. We hypothesized that a stomatal response to RL and C_a_ requires a close and direct pathway between photosynthetic mesophyll cells and the guard cells, but that the guard cell‐specific BL stomatal response will occur regardless of mesophyll anatomy.

## Materials and Methods

### Plant materials

Six representative species of Restionaceae were selected: three from Restionoideae, including *Elegia elephantina* H.P.Linder, *Elegia capensis* (Burm.f.) Schelpe, and *Restio similis* Pillans; and three from Leptocarpoideae, including *Apodasmia similis* (Edgar) B.G.Briggs & L.A.S.Johnson, *Leptocarpus roycei* B.G.Briggs, and *Leptocarpus scariosus* R.Br. All individuals except the *Leptocarpus* species were grown in pots containing a 1 : 1 : 1 mix of commercial potting mix, sand, and Indiana Miami topsoil in the glasshouses of Purdue University, West Lafayette, IN, USA under controlled conditions. Plants received daily watering and a weekly application of complete liquid fertilizer (Jack's Classic Petunia FeED, 20‐6‐22 N‐P‐K, JR Peters Inc., Allentown, PA, USA). Temperatures in the glasshouse was set at 23°C during the day and 18°C at night, and plants were grown under a natural photoperiod. *Leptocarpus* plants were grown in daily‐watered potting mix consisting of sand, lime, cocopeat, and pine mulch in a glasshouse on a 14‐h photoperiod with daytime temperatures set at 26°C and nighttime temperatures at 23°C at the University of Western Australia.

### Gas exchange measurements

Gas exchange measurements were performed using an infrared gas analyzer (Li‐6800; Li‐COR Biosciences, Lincoln, NE, USA). Before measurements were recorded, plants were kept in the dark overnight to ensure complete stomatal closure. The following morning, before sunrise, a fully developed culm was enclosed in the 6 cm^2^ cuvette of the gas analyzer, in which controlled environmental conditions were maintained: 400 μmol mol^−1^ C_a_, 23°C air temperature, a flow rate of 600 μmol m^−2^ s^−1^, and VPD of 2.4 kPa. All samples were held under dark conditions for 10 min to stabilize stomatal conductance (*g*_s_) and ensure stomatal closure. Subsequently, photosynthetic photon flux density was set to different light intensities and fluences in a sequence of (1) 600 μmol m^−2^ s^−1^ RL; followed by (2) 400 μmol m^−2^ s^−1^ BL with 600 μmol m^−2^ s^−1^ RL; then (3) 400 μmol m^−2^ s^−1^ BL; and finally (4) darkness. The duration of each transition in light was set based on the time for a stomatal response to occur, or not, and for stomata to reach a new steady state conductance.

Using *E. elephantina* and *A. similis*, as representatives of Restionoideae and Leptocarpoideae, respectively, we further investigated whether stomata responded to C_a_ as well as a higher intensity of RL. Plants were exposed to a low C_a_ of 100 μmol mol^−1^ and two cycles of 1000 μmol m^−2^ s^−1^ of RL followed by darkness, which was followed by exposure to ambient C_a_ of 400 μmol mol^−1^ under 1000 μmol m^−2^ s^−1^ of RL. In *E. elephantina*, the stomatal response to changes in the sequence of C_a_ (100, 400, 600, 1000, 100, and 600 μmol mol^−1^) from darkness to 600 and 400 μmol m^−2^ s^−1^ of RL and BL, respectively, was investigated. Gas exchange values were normalized by culm area in the cuvette, which was measured postexperiment. As all species had a cylindrical culm, the diameter was measured at both ends of the cuvette using a digital Vernier caliper, and the surface area of a frustum was calculated.

For comparing the *g*_s_ response between dark and RL, a total of three measurements were recorded for each plant species, except in *L. roycei*, in which only one replicate was recorded due to limited material.

### Mesophyll anatomy

Fresh culm sections (< 20 μm) mounted in water of all species were prepared using a microtome (Microm HM 430; Thermo Fisher Scientific, Runcorn, UK) and imaged under an epifluorescence Zeiss microscope (AxioImagerA2, Zeiss, Gottingen, Germany) using a longpass filter (365 nm excitation, 420 nm emission) to capture the autofluorescence of Chl and lignin. The same culms in which gas exchange was measured were used for anatomy, with the central region of the internode sampled. A total of 30 stomata were imaged for each species to confirm substomatal and mesophyll anatomy. In all Restionoideae species, Fiji software (Schindelin *et al*., 2012) was used to determine the volume of the substomatal cavity, assuming it was cylindrical with length and radius determined from cross‐sections.

## Results

### No stomatal response to RL when protective cells are present

**Fig. 1:**
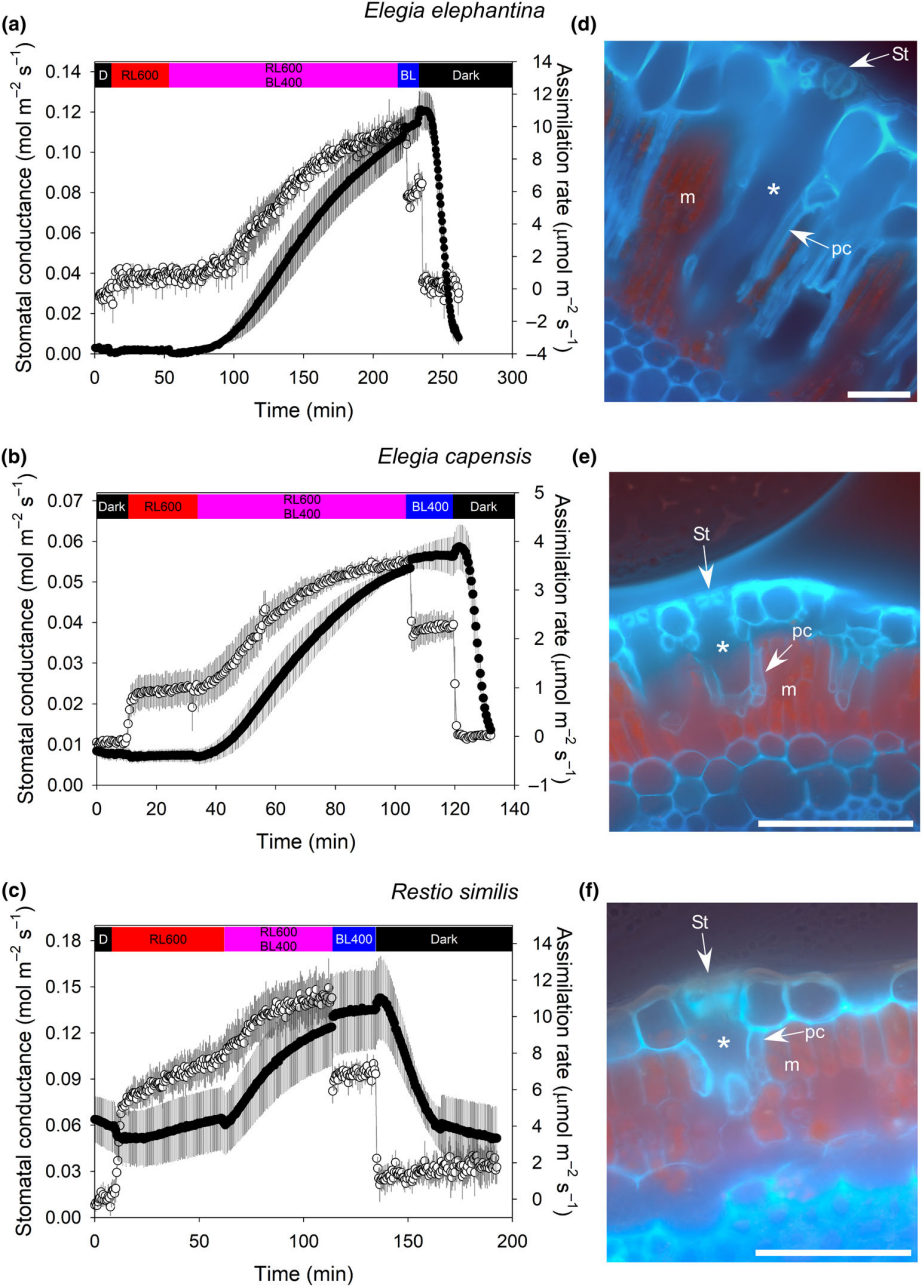
Stomata of Restionoideae species do not respond to red light (RL) and have large substomatal cavities lined with protective cells. Mean (*n* = 3, ±SE) response of stomatal conductance (black) and assimilation rate (white) in (a) *Elegia elephantina*, (b) *Elegia capensis*, and (c) *Restio similis* to step changes in light intensity and fluence from darkness to 600 μmol m^−2^ s^−1^ RL, followed by the addition of 400 μmol m^−2^ s^−1^ of blue light (BL), then the removal of RL and exposure to only BL, then darkness. The bar above the charts denotes the period of respective light exposure, where D in the black bar denotes darkness, the red bar denotes RL, the purple bar denotes mixed RL and BL, the blue bar denotes BL, and the numbers indicate photosynthetic photon flux density in μmol m^−2^ s^−1^. Fluorescent micrographs of cross‐sections through stomata (St) and substomatal chambers (denoted by an asterisk) are shown for (d) *E. elephantina*, (e) *E. capensis*, and (f) *R. similis*; protective cells (pc) and mesophyll (m) are indicated. Bars, 50 μm.

In the Restionoideae species, stomata did not open in response to 600 μmol m^−2^ s^−1^ of RL from darkness despite this RL increasing assimilation (Fig. 1). In *E. elephantina*, mean (*n* = 3, ±SE) *g*_s_ was 1.8 ± 0.9 mmol m^−2^ s^−1^ under darkness and 1.9 ± 0.9 mmol m^−2^ s^−1^ after 50 min of RL (Fig. 1a). Similarly, in the two other Restionoideae species with substomatal cavities and protective cells, *E. capensis* and *R. similis*, stomata did not open from darkness to 600 μmol m^−2^ s^−1^ of RL (Fig. 1b,c). When 400 μmol m^−2^ s^−1^ of BL was added to the previously applied RL in all Restionoideae species, *g*_s_ started to increase; to 107 ± 10 mmol m^−2^ s^−1^ in *E. elephantina;* to 53.4 ± 1 mmol m^−2^ s^−1^ in *E. capensis*; and to 128.3 ± 25 mmol m^−2^ s^−1^ in *R. similis* (Fig. 1). Removal of the RL so that culms were only exposed to 400 μmol m^−2^ s^−1^ of BL did not affect the trajectory of *g*_s_, with stomata not closing in all three Restionoideae species. In all cases when RL was removed, assimilation immediately declined to a lower rate (Fig. 1). Finally, removing the BL in all Restionoideae species triggered a decrease in g_s_ as stomata closed (Fig. 1). All three Restionoideae species had large volume substomatal cavities lined by protective cells (Fig. 1d–f), mean (*n* = 30, ±SE) substomatal cavity volumes ranged from 4.65 ± 0.32 pl in *E. capensis*, 8.42 ± 0.72 pl in *R. similis*, to 246.85 ± 17.8 pl in *E. elephantina*.

**Fig. 2:**
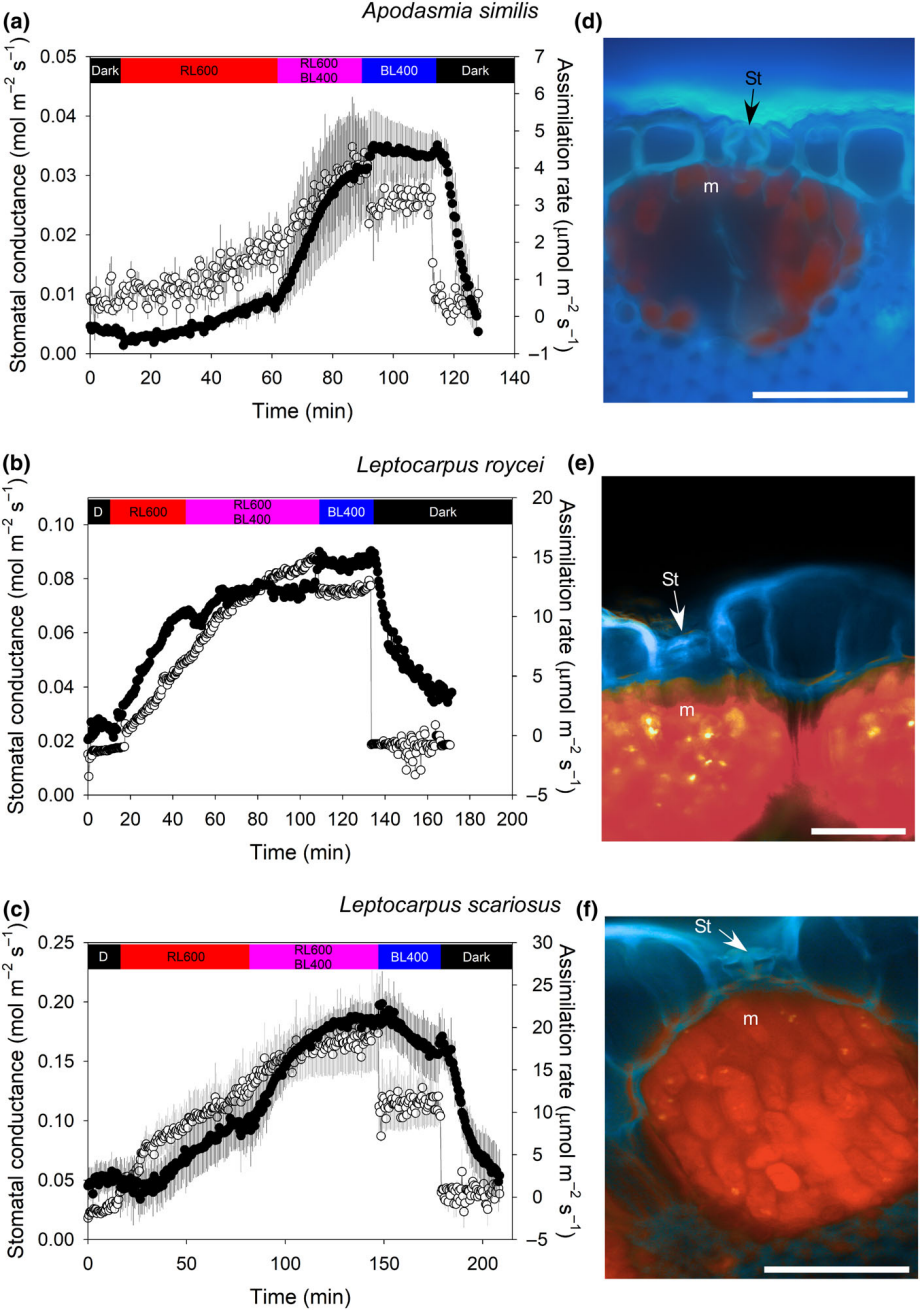
Stomata of Leptocarpoideae species respond to red light (RL) and have small substomatal cavities lined with chlorenchyma. Mean (*n* = 3, ±SE) response of stomatal conductance (black) and assimilation rate (white) in (a) *Apodasmia similis*, (b) a single trace in *Leptocarpus roycei*, and (c) mean response in *Leptocarpus scariosus* to step changes in light intensity and fluence from darkness to 600 μmol m^−2^ s^−1^ RL, followed by the addition of 400 μmol m^−2^ s^−1^ of blue light (BL), then the removal of RL and exposure to only BL, then darkness. The bar above the charts denotes the period of respective light exposure, where D in the black bar denotes dark, the red bar denotes RL, the purple bar denotes mixed RL and BL, the blue bar denotes BL, and the numbers indicate photosynthetic photon flux density in μmol m^−2^ s^−1^. Fluorescent micrographs of cross‐sections through stomata (St) and subtending mesophyll (m, red autofluorescence of Chl) are shown for (d) *A. similis*, (e) *L. roycei*, and (f) *L. scariosus*. Bars, 50 μm.

In contrast to Restionoideae species, the Leptocarpoideae species had no protective cells lining the substomatal cavity, with mesophyll cells subtending the stomata (Fig. 2d–f). Leptocarpoideae species had stomata that opened under 600 μmol m^−2^ s^−1^ of RL from darkness. In *A. similis*, g_s_ increased from 3.0 ± 0.2 to 11.0 ± 0.5 mmol m^−2^ s^−1^ in response to a transition from darkness to 600 μmol m^−2^ s^−1^ RL (Fig. 2a). Stomata showed a more pronounced RL response from darkness in *L. roycei* and *L. scariosus*, with stomata under RL opening to 67 mmol m^−2^ s^−1^ and 98 ± 21 mmol m^−2^ s^−1^, respectively (Fig. 2b,c), and in both of these species, assimilation rate increased on exposure to RL. Upon adding 400 μmol m^−2^ s^−1^ of BL to the 600 μmol m^−2^ s^−1^ of RL, *g*_s_ further increased in all Leptocarpoideae species, with *g*_s_ increasing in *A. similis* to 31.0 ± 0.8 mmol m^−2^ s^−1^, to 75 mmol m^−2^ s^−1^ in *L. roycei*, and to 185 ± 15 mmol m^−2^ s^−1^ in *L. scariosus* (Fig. 2a–c). In all Leptocarpoideae species, stomata closed in the dark, while there was variation in the response of stomata to the removal of RL when irradiated with both RL and BL, with the lower light intensity under only BL causing stomata conductance to decline in *L. scariosus* (Fig. 2c), while stomata remained open in *A. similis* and *L. roycei* (Fig. 2a). The removal of RL when irradiated with both RL and BL in *L. scariosus* resulted in the largest decline in assimilation (Fig. 2c).

### No stomatal response to CO_2_ when protective cells are present

**Fig. 3:**
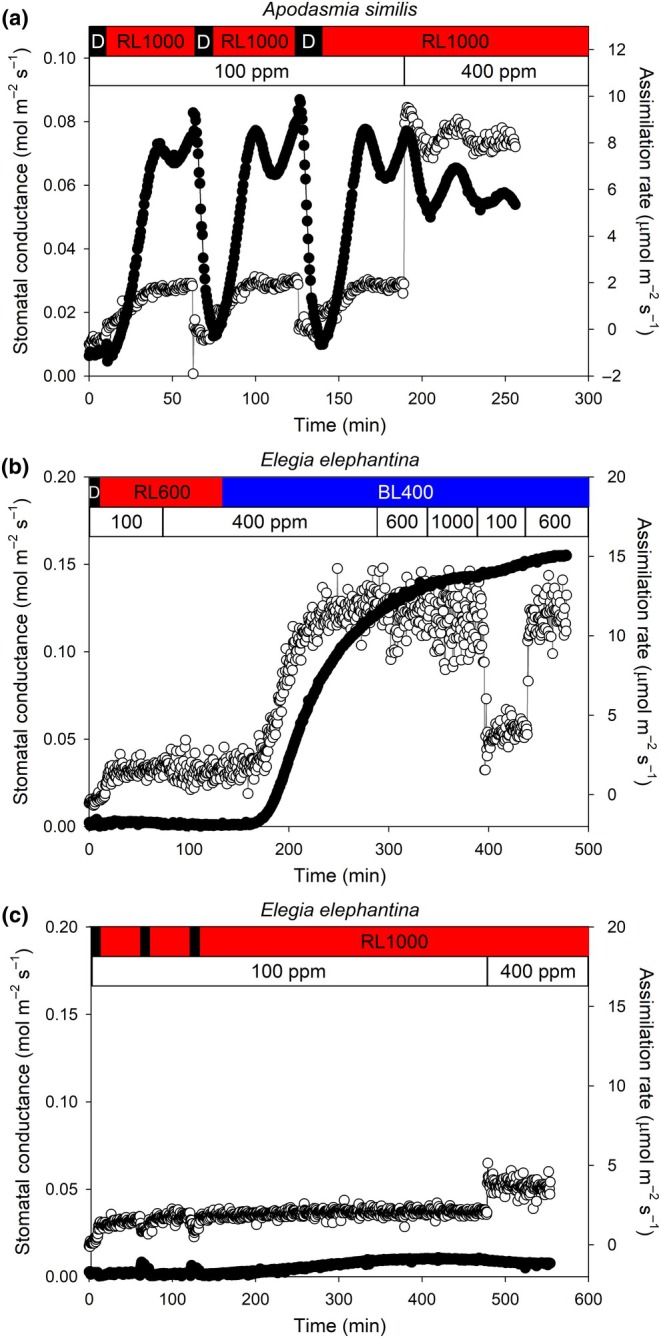
Stomata are sensitive to red light (RL) and atmospheric CO_2_ changes in *Apodasmia similis* but not in *Elegia elephantina*. (a) The response of stomatal conductance (black) and assimilation rate (white) in *A. similis* to two cycles of darkness to 1000 μmol m^−2^ s^−1^ of RL to darkness under a C_a_ of 100 μmol mol^−1^, after which under 1000 μmol m^−2^ s^−1^ of RL, C_a_ was raised to 400 μmol mol^−1^. (b) The response of *E. elephantina* stomata to a sequence of darkness to 600 μmol m^−2^ s^−1^ of RL followed by 400 μmol m^−2^ s^−1^ of only BL; during this time, C_a_ was changed (thin black line). (c) The response of *E. elephantina* stomata to two cycles of darkness to 1000 μmol m^−2^ s^−1^ of RL to darkness under a C_a_ (thin black line) of 100 μmol mol^−1^, after which under a constant 1000 μmol m^−2^ s^−1^ of RL C_a_ was raised to 400 μmol mol^−1^. The top bar above the charts qqdenotes the period of respective light exposure, where black denotes dark, red denotes RL, blue denotes BL, and the letters and numbers indicate either darkness (d) or light color as well as photosynthetic photon flux density in μmol m^−2^ s^−1^. The lower bar at the top of the charts denotes C_a_.

Using *E. elephantina* as a representative species of the subfamily Restionoideae and *A. similis* of the subfamily Leptocarpoideae, we further investigated whether stomata responded to changes in C_a_, as well as whether a higher RL under low C_a_ could open stomata. In *A. similis*, *g*_s_ responded rapidly to increases in 1000 μmol m^−2^ s^−1^ of RL under low C_a_ from 6.3 to 70 mmol m^−2^ s^−1^ within 30 min (Fig. 3a), which was faster and more pronounced than stomatal opening under lower fluences of RL at ambient C_a_ (Fig. 2a). In *E. elephantina*, stomata under BL did not respond to increases or decreases in atmospheric C_a_, with stomata remaining open and unresponsive to increases up to 1000 μmol mol^−1^ CO_2_ (Fig. 3b). Unlike in *A. similis*, the stomata of *E. elephantina* did not close when C_a_ was raised from 100 μmol mol^−1^ to 600 μmol mol^−1^ (Fig. 3a,b). In *E. elephantina*, exposure to 100 μmol mol^−1^ CO_2_ in the dark did not induce stomatal opening, and when irradiated with 1000 μmol m^−2^ s^−1^ of RL under low C_a_, *g*_s_ increased gradually from 1 mmol m^−2^ s^−1^ which was lowest in the dark, to a maximum of only 10.9 mmol m^−2^ s^−1^ over 419.5 min (Fig. 3c).

## Discussion

### Substomatal anatomy evolution and stomatal response to light

Restionaceae species of subfamily Restionoideae with protective cells below the stomata (Linder, 2000; Linder & Hardy, 2010; Briggs *et al*., 2014) do not have a stomatal response to RL or C_a_, instead opening only in response to BL (Figs 1 and 3). By contrast, the stomata of representative species from subfamily Leptocarpoideae, which lack protective cells lining the substomatal cavity, are responsive to RL, BL, and C_a_ (Figs 2 and 3). In the Leptocarpoideae species, *g*_s_ was lower in RL, increasing with the addition of BL, which is consistent with other studies in angiosperms (Assmann, 1988; Kinoshita & Hayashi, 2011; Yang *et al*., 2020; Vialet‐Chabrand *et al*., 2021; Bernardo *et al*., 2023; Li *et al*., 2023). The lack of a stomatal response to RL in species of Restionoideae appears to be linked to the presence of protective cells that do not contain chloroplasts and have a cuticle (Fig. 1) because they presumably block a photosynthetic mesophyll signal from reaching guard cells. The presence of protective cells is a phylogenetically relevant trait in Restionaceae (Linder, 2000; Briggs & Linder, 2009), long speculated to be an adaptation to xeric environments (Gilg, 1891; Moline & Linder, 2005; Linder & Hardy, 2010; Briggs *et al*., 2014), although attributing protective cells to aridity has also long been questioned (Pfitzer, 1870). Recent work suggests that despite being native to high irradiance environments that experience long periods of drought, many species of Restionoideae have highly vulnerable xylem, remain at hydrated water potentials, and maintain gas exchange through low rainfall periods in the summer (Skelton *et al*., 2023; West *et al*., 2024). Hydration and summer gas exchange are maintained by harvesting dew on the culms (West *et al*., 2012; Skelton *et al*., 2023). This dew‐harvesting ecology may not occur in Australian Restionaceae; with the morphologically similar and closely related *Lyginia barbarta* R.Br. (Anarthraceae) from Western Australia having greatly reduced gas exchange during the low rainfall summer, with hydration during this time supplied by soil moisture from deep layers (Shane *et al*., 2010). Our results suggest that in a dew‐covered culm, the stomata of species of Restionoideae without a mesophyll photosynthetic feedback signal could open with increasing BL, even if there is no chance of CO_2_ exchange for photosynthesis – this might allow plants to maximize gas exchange as soon as dew evaporates. More work is required to associate protective cells with the harvesting of dew from culms. A unique feature of the anatomy of Leptocarpoideae species is the presence of pillar cells that extend from the epidermis to the pith of the culm, creating isolated pockets of mesophyll (Linder, 2000), whether this isolation of mesophyll by pillar cells influences stomatal responses to light remains unknown.

Protective cells in Restionoideae may not be a drought‐tolerance adaptation but rather limit a mesophyll photosynthetic feedback signal from influencing stomatal aperture, resulting in the absence of this stomatal regulation. It could be that an absence of a stomatal response to RL ensures stomata are only regulated by BL. Levels of BL are highest in direct sun, like the highlight environments of the oligotrophic heaths to which Restionaceae are native (Linder, 1987; Jamieson & Kirstenbosch, 1994); under these conditions, stomatal regulation by RL might not be as important. Although most Australian Restionaceae without protective cells inhabit similar environments (Briggs *et al*., 2014). Comparisons of stomatal responses and drought physiology across Restionaceae, including species from the Australian subfamily Sporodanthoideae and species of the *Desmocladus* clade within Leptocarpoideae, which have convergently evolved protective cells (Linder, 2000), would be helpful for resolving questions about the adaptive relevance of protective cells and large substomatal chambers across Restionaceae. In addition, there are reports of a number of species in closely related families to Restionaceae that share similar internal anatomies with substomatal cavities lined with unique cells resembling protective cells (Firbas, 1931) as well as some cycad species in Zamiaceae where cells lining the substomatal cavity have secondarily thickened cell walls (Coiro & Pott, 2017). These species could be highly informative for resolving the selective pressure that drove the evolution of protective cells, the elimination of stomatal responses to RL and C_a_ and presumably all photosynthetic feedback signals from the mesophyll.

### The mesophyll signal for RL responses

Our results show that a mesophyll‐derived signal that triggers stomatal responses to RL and C_a_ (Matthews *et al*., 2019) is unable to traverse the protective cells and reach the guard cells. This has implications for understanding the mechanism of the mesophyll feedback signal and stomatal control by RL in angiosperms. Our results suggest that photosynthesis in guard cells, which has been suggested to be a driver of stomatal responses to RL in angiosperms (Lawson *et al*., 2003; Lawson & Blatt, 2014), is not the primary driver of this stomatal response in Restionaceae. We also find that in the absence of a mesophyll feedback signal, the stomata of Restionoideae appear relatively insensitive to changes in assimilation rate driven by changes in the intensity of RL (Fig. 1). It is not known the extent to which protective cells might limit the internal diffusion of CO_2_, although we did measure relatively high photosynthetic rates in some of the Restionoideae species (Fig. 1), which suggests minimal internal limitations to CO_2_ diffusion when protective cells are present.

Our results confirm observations from epidermal peel studies that indicate a direct contact between guard cells and photosynthetic mesophyll is essential for stomatal responses to C_a_ (Mott, 2009). Protective cells have a cuticle (Botha *et al*., 1972; Yates, 2011) but do not completely enclose the substomatal chamber; there are gaps between the cells at the end furthest from the guard cells (Pfitzer, 1870). Given that there is a continuous vapor connection between guard cells and mesophyll, albeit across a long distance, it appears unlikely that this signal is a vapor (Sibbernsen & Mott, 2010; Mott & Peak, 2013), although more work is required to resolve this. Other studies have suggested that the mesophyll feedback signal is in an aqueous form (Hedrich & Maten, 1993; Hedrich *et al*., 1994; Tominaga *et al*., 2001; Lee *et al*., 2008; Fujita *et al*., 2013, 2019), with recent work suggesting that this signal is most likely apoplastic sugar (Flütsch & Santelia, 2021; Zait *et al*., 2025). We speculate that apoplastic sugar is not reaching the guard cells in the species with protective cells. We do not know the apoplastic pathway for guard cell water supply, but given the cuticle covering on the substomatal chamber side of the protective cells (Botha *et al*., 1972; Yates, 2011), it seems most likely that apoplastic water reaches the guard cells in species of Restionoideae via the epidermis. Whether hypodermal anatomy influences this apoplastic water transport to the guard cells remains unknown. It is also not known whether an increase in transpiration, for example, at a higher VPD (the experiments here were conducted at 2.4 kPa), could increase apoplastic transport and restore RL and CO_2_ signalling in Restionoideae species.

### Conclusion

Here we used the highly divergent substomatal anatomy of Restionaceae, with species from the subfamily Restionoideae having a substomatal chamber lined with protective cells, and representative species from the subfamily Leptocarpoideae having a substomatal chamber lined with mesophyll cells, to investigate whether substomatal anatomy influences stomatal responsiveness to light wavelengths and C_a_. The presence of protective cells lining the substomatal chamber inhibited stomatal responses to RL and C_a_ in Restionoideae species, with the light responses of these stomata being driven only by BL. These results suggest that protective cells block a mesophyll feedback signal from regulating stomata. Our study provides new insight into how mesophyll anatomy can regulate stomatal response to light and C_a_ and suggests that the mesophyll feedback signal for stomatal regulation requires the close proximity of guard cells to chlorenchyma.

## Competing interests

None declared.

## Author contributions

SAMM devised the project with help from AM, FS and MH, MH and CIF collected data; MH analyzed data and wrote the manuscript with SAMM. All authors contributed to editing the manuscript.

## Disclaimer

The New Phytologist Foundation remains neutral with regard to jurisdictional claims in maps and in any institutional affiliations.

## Data Availability

All data are included in the manuscript.

## References

[nph70667-bib-0001] Ando E , Kinoshita T . 2018. Red light‐induced phosphorylation of plasma membrane H^+^‐ATPase in stomatal guard cells. Plant Physiology 178: 838–849. 30104254 10.1104/pp.18.00544 PMC6181031

[nph70667-bib-0002] Assmann SM . 1988. Enhancement of the stomatal response to blue light by red light, reduced intercellular concentrations of CO_2_, and low vapor pressure differences. Plant Physiology 87: 226–231. 16666108 10.1104/pp.87.1.226 PMC1054730

[nph70667-bib-0003] Bernardo EL , Sales CRG , Cubas LA , Vath RL , Kromdijk J . 2023. A comparison of stomatal conductance responses to blue and red light between C_3_ and C_4_ photosynthetic species in three phylogenetically‐controlled experiments. Frontiers in Plant Science 14: 1253976. 37828928 10.3389/fpls.2023.1253976 PMC10565490

[nph70667-bib-0004] Borsuk AM , Roddy AB , Théroux‐Rancourt G , Brodersen CR . 2022. Structural organization of the spongy mesophyll. New Phytologist 234: 946–960. 35037256 10.1111/nph.17971 PMC9303971

[nph70667-bib-0005] Botha DJ , Van der Schijff HP , Van Tonder EMA . 1972. The position, structure and ontogeny of the stomata in the stems of *Elegia vaginulata* Mast. M.Sc. thesis, University of Pretoria.

[nph70667-bib-0006] Briggs BG , Linder HP . 2009. A new subfamilial and tribal classification of Restionaceae (Poales). Telopea 12: 333–345.

[nph70667-bib-0007] Briggs BG , Marchant AD , Perkins AJ . 2014. Phylogeny of the restiid clade (Poales) and implications for the classification of Anarthriaceae, Centrolepidaceae and Australian Restionaceae. Taxon 63: 24–46.

[nph70667-bib-0008] Coiro M , Pott C . 2017. *Eobowenia* gen. nov. from the Early Cretaceous of Patagonia: indication for an early divergence of *Bowenia*? BMC Evolutionary Biology 17: 97. 28388891 10.1186/s12862-017-0943-x PMC5383990

[nph70667-bib-0009] Cutler DF . 1969. In: Metcalf CR , ed. Anatomy of the Monocotyledons. Volume IV Juncales. Oxford, MI, USA: Oxford University Press.

[nph70667-bib-0071] Doi M , Kitagawa Y , Shimazaki K . 2015. Stomatal blue light response is present in early vascular plants. Plant Physiology 169: 1205–1213. 26307440 10.1104/pp.15.00134 PMC4587438

[nph70667-bib-0010] Firbas F . 1931. Untersuchungen über den Wasserhaushalt der Hochmoorpflanzen. Jahrbucher fur Wissenschaftliche Botanik 74: 457–696.

[nph70667-bib-0070] Flütsch S , Santelia D . 2021. Mesophyll‐derived sugars are positive regulators of light‐driven stomatal opening. New Phytologist 230: 1754–1760. 33666260 10.1111/nph.17322

[nph70667-bib-0011] Fujita T , Noguchi K , Ozaki H , Terashima I . 2019. Confirmation of mesophyll signals controlling stomatal responses by a newly devised transplanting method. Functional Plant Biology 46: 467–481. 30940335 10.1071/FP18250

[nph70667-bib-0012] Fujita T , Noguchi K , Terashima I . 2013. Apoplastic mesophyll signals induce rapid stomatal responses to CO_2_ in *Commelina communis* . New Phytologist 199: 395–406. 23560389 10.1111/nph.12261

[nph70667-bib-0013] Gilg E . 1891. Beiträge zur vergleichenden Anatomie der xerophilen Familie der Restiaceae. Friedrich Wilhelm Universität von Berlin; Dissertation.

[nph70667-bib-0014] Grantz DA , Zeiger E . 1986. Stomatal responses to light and leaf‐air water vapor pressure difference show similar kinetics in sugarcane and soybean. Plant Physiology 81: 865–868. 16664916 10.1104/pp.81.3.865 PMC1075441

[nph70667-bib-0015] Hedrich R , Marten I , Lohse G , Dietrich P , Winter H , Lohaus G , Haldt H‐W . 1994. Malate‐sensitive anion channels enable guard cells to sense changes in the ambient CO_2_ concentration. The Plant Journal 6: 741–748.

[nph70667-bib-0016] Hedrich R , Maten I . 1993. Malate‐induced feedback regulation of plasma membrane anion channels could provide a CO_2_ sensor to guard cells. EMBO Journal 12: 897–901. 7681395 10.1002/j.1460-2075.1993.tb05730.x PMC413288

[nph70667-bib-0017] Hiyama A , Takemiya A , Munemasa S , Okuma E , Sugiyama N , Tada Y , Murata Y , Shimazaki KI . 2017. Blue light and CO_2_ signals converge to regulate light‐induced stomatal opening. Nature Communications 8: 1–13. 10.1038/s41467-017-01237-5 PMC5670223 29101334

[nph70667-bib-0018] Inoue SI , Kinoshita T . 2017. Blue light regulation of stomatal opening and the plasma membrane H+‐ATPase. Plant Physiology 174: 531–538. 28465463 10.1104/pp.17.00166 PMC5462062

[nph70667-bib-0019] Inoue SI , Kinoshita T , Matsumoto M , Nakayama KI , Doi M , Shimazaki KI . 2008. Blue light‐induced autophosphorylation of phototropin is a primary step for signaling. Proceedings of the National Academy of Sciences, USA 105: 5626–5631. 10.1073/pnas.0709189105 PMC2291087 18378899

[nph70667-bib-0020] Ivsic C , Shabala S , Sussmilch FC . 2025. Evolutionary insights into light‐induced stomatal opening mechanisms. Trends in Plant Science 30: 886–896. 40222890 10.1016/j.tplants.2025.03.005

[nph70667-bib-0021] Jamieson H , Kirstenbosch BN . 1994. The restio garden at Kirstenbosch. Veld and Flora 80: 124–125.

[nph70667-bib-0022] Kinoshita T , Hayashi Y . 2011. New insights into the regulation of stomatal opening by blue light and plasma membrane H+‐ATPase. International Review of Cell and Molecular Biology 289: 89–115. 21749899 10.1016/B978-0-12-386039-2.00003-1

[nph70667-bib-0023] Lawson T , Blatt MR . 2014. Stomatal size, speed, and responsiveness impact on photosynthesis and water use efficiency. Plant Physiology 164: 1556–1570. 24578506 10.1104/pp.114.237107 PMC3982722

[nph70667-bib-0024] Lawson T , Leakey ADB . 2024. Stomata: custodians of leaf gaseous exchange. Journal of Experimental Botany 75: 6677–6682. 39545386 10.1093/jxb/erae425 PMC11565196

[nph70667-bib-0025] Lawson T , Matthews J . 2020. Guard cell metabolism and stomatal function. Annual Review of Plant Biology 71: 273–302. 10.1146/annurev-arplant-050718-100251 32155341

[nph70667-bib-0026] Lawson T , Oxborough K , Morison JIL , Baker NR . 2003. The responses of guard and mesophyll cell photosynthesis to CO_2_, O_2_, light, and water stress in a range of species are similar. Journal of Experimental Botany 54: 1743–1752. 12773521 10.1093/jxb/erg186

[nph70667-bib-0027] Lawson T , Vialet‐Chabrand S . 2019. Speedy stomata, photosynthesis and plant water use efficiency. New Phytologist 221: 93–98. 29987878 10.1111/nph.15330

[nph70667-bib-0028] Lee J , Bowling DJF . 1992. Effect of the mesophyll on stomatal opening in *Commelina communis* . Journal of Experimental Botany 43: 951–957.

[nph70667-bib-0029] Lee J , Bowling DJF . 1995. Influence of the mesophyll on stomatal opening. Functional Plant Biology 22: 357–363.

[nph70667-bib-0030] Lee M , Choi Y , Burla B , Kim Y‐Y , Jeon B , Maeshima M , Yoo J‐Y , Martinoia E , Lee Y . 2008. The ABC transporter AtABCB14 is a malate importer and modulates stomatal response to CO_2_ . Nature Cell Biology 10: 1217–1223. 18776898 10.1038/ncb1782

[nph70667-bib-0031] Li X , Zhao S , Lin A , Yang Y , Zhang G , Xu P , Wu Y , Yang Z . 2023. Effect of different ratios of red and blue light on maximum stomatal conductance and response rate of cucumber seedling leaves. Agronomy 13: 19–41.

[nph70667-bib-0032] Linder HP . 1987. The evolutionary history of the Poales/Restionales: a hypothesis. Kew Bulletin 42: 297.

[nph70667-bib-0033] Linder HP . 2000. Vicariance, climate change, anatomy and phylogeny of Restionaceae. Botanical Journal of the Linnean Society 134: 159–177.

[nph70667-bib-0034] Linder HP , Hardy CR . 2010. A generic classification of the Restioneae (Restionaceae), southern Africa. Bothalia 40: 1–35.

[nph70667-bib-0035] Liu J , van Iersel MW . 2021. Photosynthetic physiology of blue, green, and red light: light intensity effects and underlying mechanisms. Frontiers in Plant Science 12: 619987. 33747002 10.3389/fpls.2021.619987 PMC7977723

[nph70667-bib-0036] Long SP , Taylor SH , Burgess SJ , Carmo‐Silva E , Lawson T , De Souza AP , Leonelli L , Wang Y . 2022. Into the shadows and back into sunlight: photosynthesis in fluctuating light. Annual Review of Plant Biology 73: 617–648. 10.1146/annurev-arplant-070221-024745 35595290

[nph70667-bib-0037] Matthews JSA , Vialet‐Chabrand S , Lawson T . 2018. Acclimation to fluctuating light impacts the rapidity of response and diurnal rhythm of stomatal conductance. Plant Physiology 176: 1939–1951. 29371250 10.1104/pp.17.01809 PMC5841698

[nph70667-bib-0038] Matthews JSA , Vialet‐Chabrand S , Lawson T . 2019. Role of blue and red light in stomatal dynamic behaviour. Journal of Experimental Botany 71: 2253. 10.1093/jxb/erz563 PMC7134916 31872212

[nph70667-bib-0039] Matthews JSA , Vialet‐Chabrand SRM , Lawson T . 2017. Diurnal variation in gas exchange: the balance between carbon fixation and water loss. Plant Physiology 174: 614–623. 28416704 10.1104/pp.17.00152 PMC5462061

[nph70667-bib-0040] McAdam SAM , Brodribb TJ . 2012. Stomatal innovation and the rise of seed plants. Ecology Letters 15: 1–8. 22017636 10.1111/j.1461-0248.2011.01700.x

[nph70667-bib-0041] McAusland L , Vialet‐Chabrand S , Davey P , Baker NR , Brendel O , Lawson T . 2016. Effects of kinetics of light‐induced stomatal responses on photosynthesis and water‐use efficiency. New Phytologist 211: 1209–1220. 27214387 10.1111/nph.14000 PMC4982059

[nph70667-bib-0042] McCree KJ . 1971. The action spectrum, absorptance and quantum yield of photosynthesis in crop plants. Agricultural Meteorology 9: 191–216.

[nph70667-bib-0043] McCree KJ . 1972. Test of current definitions of photosynthetically active radiation against leaf photosynthesis data. Agricultural Meteorology 10: 443–453.

[nph70667-bib-0044] Moline PM , Linder HP . 2005. Molecular phylogeny and generic delimitation in the *Elegia* GROUP (Restionaceae, South Africa) based on a complete taxon sampling and four chloroplast DNA regions. Systematic Botany 30: 759–772.

[nph70667-bib-0068] Mott KA , Sibbernsen ED , Shope JC . 2008. The role of the mesophyll in stomatal responses to light and CO_2_ . Plant, Cell and Environment 31: 1299–1306. 10.1111/j.1365-3040.2008.01845.x 18541006

[nph70667-bib-0045] Mott K . 2009. Opinion: Stomatal responses to light and CO_2_ depend on the mesophyll. Plant, Cell & Environment 32: 1479–1486. 10.1111/j.1365-3040.2009.02022.x 19627565

[nph70667-bib-0046] Mott K , Peak D . 2013. Testing a vapour‐phase model of stomatal responses to humidity. Plant, Cell & Environment 36: 936–944. 10.1111/pce.12026 23072325

[nph70667-bib-0047] Pfitzer E . 1870. Beiträge zur Kenntnis der Hautgewebe der Pflanzen. Jahrbucher fur Wissenschaftliche Botanik 7: 561–581.

[nph70667-bib-0048] Raschke K . 1975. Stomatal action. Annual Review of Plant Biology 26: 309–340.

[nph70667-bib-0049] Roelfsema MRG , Hanstein S , Felle HH , Hedrich R . 2002. CO_2_ provides an intermediate link in the red light response of guard cells. The Plant Journal 32: 65–75. 12366801 10.1046/j.1365-313x.2002.01403.x

[nph70667-bib-0050] Santos MG , Davey PA , Hofmann TA , Borland A , Hartwell J , Lawson T . 2021. Stomatal responses to light, CO_2_, and mesophyll tissue in *Vicia faba* and *Kalanchoë fedtschenkoi* . Frontiers in Plant Science 12: 740534. 34777422 10.3389/fpls.2021.740534 PMC8579043

[nph70667-bib-0051] Schindelin J , Arganda‐Carreras I , Frise E , Kaynig V , Longair M , Pietzsch T , Preibisch S , Rueden C , Saalfeld S , Schmid B *et al*. 2012. Fiji: an open‐source platform for biological‐image analysis. Nature Methods 9: 676–682. 22743772 10.1038/nmeth.2019 PMC3855844

[nph70667-bib-0052] Schwartz A , Zeiger E . 1984. Metabolic energy for stomatal opening. Roles of photophosphorylation and oxidative phosphorylation. Planta 161: 129–136. 24253600 10.1007/BF00395472

[nph70667-bib-0053] Shane MW , McCully ME , Canny MJ , Pate JS , Huang C , Ngo H , Lambers H . 2010. Seasonal water relations of *Lyginia barbata* (Southern rush) in relation to root xylem development and summer dormancy of root apices. New Phytologist 185: 1025–1037. 20085620 10.1111/j.1469-8137.2009.03143.x

[nph70667-bib-0054] Shimazaki KI , Doi M , Assmann SM , Kinoshita T . 2007. Light regulation of stomatal movement. Annual Review of Plant Biology 58: 219–247. 10.1146/annurev.arplant.57.032905.105434 17209798

[nph70667-bib-0055] Sibbernsen E , Mott KA . 2010. Stomatal responses to flooding of the intercellular air spaces suggest a vapor‐phase signal between the mesophyll and the guard cells. Plant Physiology 153: 1435–1442. 20472750 10.1104/pp.110.157685 PMC2899896

[nph70667-bib-0056] Skelton RP , West AG , Buttner D , Dawson TE . 2023. Consistent responses to moisture stress despite diverse growth forms within mountain fynbos communities. Oecologia 201: 323–339. 36692692 10.1007/s00442-023-05326-9 PMC9944370

[nph70667-bib-0057] Talbott LD , Rahveh E , Zeiger E . 2003. Relative humidity is a key factor in the acclimation of the stomatal response to CO_2_ . Journal of Experimental Botany 54: 2141–2147. 12867546 10.1093/jxb/erg215

[nph70667-bib-0058] Taylor G , Walter J , Kromdijk J . 2024. Illuminating stomatal responses to red light: establishing the role of Ci‐dependent versus ‐independent mechanisms in control of stomatal behaviour. Journal of Experimental Botany 75: 6810–6822. 38442206 10.1093/jxb/erae093 PMC11565200

[nph70667-bib-0059] Tominaga M , Kinoshita T , Shimazaki KI . 2001. Guard‐cell chloroplasts provide ATP required for H+ pumping in the plasma membrane and stomatal opening. Plant & Cell Physiology 42: 795–802. 11522904 10.1093/pcp/pce101

[nph70667-bib-0060] Vialet‐Chabrand S , Matthews JSA , Lawson T . 2021. Light, power, action! Interaction of respiratory energy‐ and blue light‐induced stomatal movements. New Phytologist 231: 2231–2246. 34101837 10.1111/nph.17538

[nph70667-bib-0061] West AG , Atkins K , van Blerk JJ , Skelton RP . 2024. Assessing vulnerability to embolism and hydraulic safety margins in reed‐like Restionaceae. Plant Biology 26: 633–646. 38588329 10.1111/plb.13644

[nph70667-bib-0062] West AG , Dawson TE , February EC , Midgley GF , Bond WJ , Aston TL . 2012. Diverse functional responses to drought in a Mediterranean‐type shrubland in South Africa. New Phytologist 195: 396–407. 22594652 10.1111/j.1469-8137.2012.04170.x

[nph70667-bib-0063] Wong SC , Cowan IR , Farquhar GD . 1979. Stomatal conductance correlates with photosynthetic capacity. Nature 282: 424–426.

[nph70667-bib-0064] Yang J , Li C , Kong D , Guo F , Wei H . 2020. Light‐mediated signaling and metabolic changes coordinate stomatal opening and closure. Frontiers in Plant Science 11: 601478. 33343603 10.3389/fpls.2020.601478 PMC7746640

[nph70667-bib-0065] Yates MJ . 2011. The functional, ecological and evolutionary significance of culm structures in the Cape Floristic Region. M.Sc. thesis, South Africa: University of Cape Town.

[nph70667-bib-0069] Zait Y , Zhu M , Ando E , Zhou Y , Yaaran A , Yon S , Okamoto M , Hayashi Y , Hirai MY , Jegla T *et al*. 2025. Apoplastic metabolomics reveals sugars as mesophyll messengers regulating guard cell ion transport under red light. Nature Plants 11: 1847–1862. 40855242 10.1038/s41477-025-02078-7 PMC12449266

[nph70667-bib-0066] Zeiger E , Zhu J . 1998. Role of zeaxanthin in blue light photoreception and the modulation of light‐CO_2_ interactions in guard cells. Journal of Experimental Botany 49: 433–442.

[nph70667-bib-0067] Zhu M , Geng S , Chakravorty D , Guan Q , Chen S , Assmann SM . 2020. Metabolomics of red‐light‐induced stomatal opening in *Arabidopsis thaliana*: coupling with abscisic acid and jasmonic acid metabolism. The Plant Journal 101: 1331–1348. 31677315 10.1111/tpj.14594

